# Transcriptional profiling of suberoylanilide hydroxamic acid (SAHA) regulated genes in mineralizing dental pulp cells at early and late time points

**DOI:** 10.1016/j.gdata.2015.07.013

**Published:** 2015-07-15

**Authors:** Henry F. Duncan, Anthony J. Smith, Garry J.P. Fleming, Gary P. Moran, Paul R. Cooper

**Affiliations:** a Division of Restorative Dentistry & Periodontology, Dublin Dental University Hospital, Trinity College Dublin, Lincoln Place, Dublin 2, Ireland; b Oral Biology, School of Dentistry, University of Birmingham, Birmingham B4 6NN, UK; c Material Science Unit, Dublin Dental University Hospital, Trinity College Dublin, Ireland; d Division of Oral Biosciences, Dublin Dental University Hospital, Trinity College Dublin, Ireland

**Keywords:** Dental pulp cells, Mineralization, Epigenetics, Histone deacetylase inhibitors, Endodontics

## Abstract

Dental pulp tissue can be damaged by a range of irritants, however, if the irritation is removed and/or the tooth is adequately restored, pulp regeneration is possible (Mjör and Tronstad, 1974 [1]). At present, dental restorative materials limit healing by impairing mineralization and repair processes and as a result new biologically-based materials are being developed (Ferracane et al., 2010 [2]). Previous studies have highlighted the benefit of epigenetic modification by histone deacetylase inhibitor (HDACi) application to dental pulp cells (DPCs), which induces changes to chromatin architecture, promoting gene expression and cellular-reparative events (Duncan et al., 2013 [3]; Paino et al., 2014 [4]). In this study a genome-wide transcription profiling in epigenetically-modified mineralizing primary DPC cultures was performed, at relatively early and late time-points, to identify differentially regulated transcripts that may provide novel therapeutic targets for use in restorative dentistry. Here we provide detailed methods and analysis on these microarray data which has been deposited in Gene Expression Omnibus (GEO): GSE67175.

**Table:** 

Specifications	
Organism/cell line/tissue	*Rattus norvegicus*/dental pulp tissue
Sex	Male
Sequencer or array type	Agilent Sure Print 4x44K array in situ oligonucleotide (G2519F)
Data format	Raw and processed
Experimental factors	Cultured primary dental pulp cells (DPCs) extirpated from the incisor teeth of Wistar Hannover rats aged 25–30 days
Experimental features	High-throughput investigation of genome-wide transcripts, epigenetically regulated by histone deacetylase inhibitors (HDACi). Four independent biological tissue samples were cultured at two different time points (24 h and 14 days) under mineralizing conditions in the presence or absence of the HDACi, SAHA.
Consent	N/A
Sample source location	University of Dublin, Dublin, Ireland, Europe

## 1 Direct link to deposited data

http://www.ncbi.nlm.nih.gov/gds/?term=GSE67175.

## 2 Experimental design, materials and methods

### 2.1 Primary cell culture

Primary DPCs were isolated from the extirpated pulp tissue of freshly extracted rodent incisor teeth using enzymatic disaggregation procedure as previously described [5]. The maxillary and mandibular incisor teeth were dissected from male Wistar Hannover rats aged 25–30 days and weighing 120–140 g. The pulp tissue was extirpated from the pulp chamber, minced with a scalpel into 1 mm^3^ pieces and transferred into Hank's balanced salt solution (Sigma-Aldrich, Arklow, Ireland) prior to incubation at 37 °C, 5% CO_2_ for 30 min (MCO-18AC incubator, Sanyo Electric, Osaka, Japan). Cells were transferred to an equal volume of supplemented α-MEM (Biosera, East Sussex, UK) containing 1% (w/v) penicillin/streptomycin (Sigma-Aldrich) and 10% (v/v) fetal calf serum (FCS) (Biosera). A single cell suspension was obtained by passing through a 70 μm cell sieve (BD Biosciences, Oxford, UK) prior to centrifugation and re-suspension in 1 ml supplemented α-MEM. DPCs were expanded under standard culture conditions to passage 2. The DPCs were seeded in supplemented α-MEM for 3 days (experimental day 0), and then either cultured for 24 h in supplemented mineralizing medium containing 1 μM SAHA prior to harvest (24 h samples) or incubated with an HDACi-free mineralizing medium for a further 13 days (14 day samples). Control samples contained cells cultured in mineralizing medium without SAHA.

### 2.2 Suberoylanilide hydroxamic acid (SAHA) preparation

A 5 mM stock solution of SAHA (*N*-hydroxy-*N*′-phenyl-octanediamide) (Sigma-Aldrich), in dimethyl sulfoxide (DMSO) was diluted in phosphate buffered saline (PBS) (Sigma-Aldrich) prior to further dilution to 1 μM with supplemented α-MEM.

### 2.3 RNA isolation, cDNA and labeled cRNA preparation

Cultures were detached (trypsin/EDTA), homogenized and RNA extracted using the RNeasy mini kit (Qiagen, West Sussex, UK), before being quantified spectrophotometrically (Nanodrop 2000, Thermo Fisher Scientific). A 75 ng aliquot of total RNA was converted to cDNA using an oligo dT-promoter primer and Affinity-Script-RT (Agilent Technologies), prior to being labeled with either Cyanine 3-CTP or Cyanine 5-CTP using the Two-Color Low Input Quick Amp labeling Kit (Agilent Technologies, Cork, Ireland) and transcribed to cRNA (Agilent Technologies). The cRNA was purified using the RNeasy mini kit (Qiagen) and Cy3, Cy5 concentration, RNA absorbance 260/280 nm and cRNA concentrations determined spectrophotometrically (Nanodrop 2000, Wilmington, DE, USA). This enabled specific activity and target yields to be calculated prior to microarray experimentation.

### 2.4 Microarray experimentation and analysis

The Agilent 4x44K v3 whole rat genome oligonucleotide gene expression microarray (Agilent Technologies) was used to analyze the transcript profiles of HDACi treated (1 μM SAHA) and untreated DPC cultures at both 24 h and 14 days under mineralizing conditions. The microarray analyses were performed on quadruplicate independent DPC cultures (n = 4) at both time-points. A total of 825 ng of labeled cRNA from each sample (treated and untreated) was loaded onto an individual array according to the manufacturer's instructions and co-hybridized at 65 °C for 17 h, washed and scanned in GenePix-Personal 4100A, Pro 6.1 (Axon, Molecular Devices, CA, USA) at a resolution of 5 μm.

**Fig. 1:**
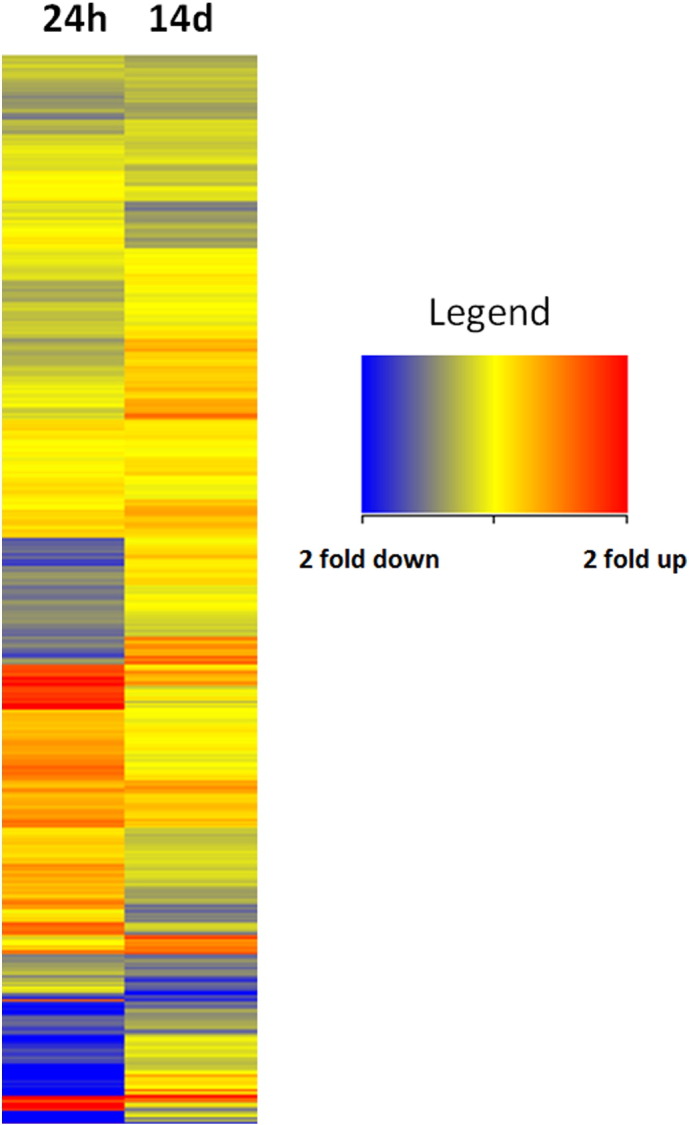
A comparison of > 2 fold gene expression at 24 h and 14 days in DPCs after exposure to SAHA for 24 h. The ‘heat-map’ shows expression patterns for all present genes.

**Fig. 2:**
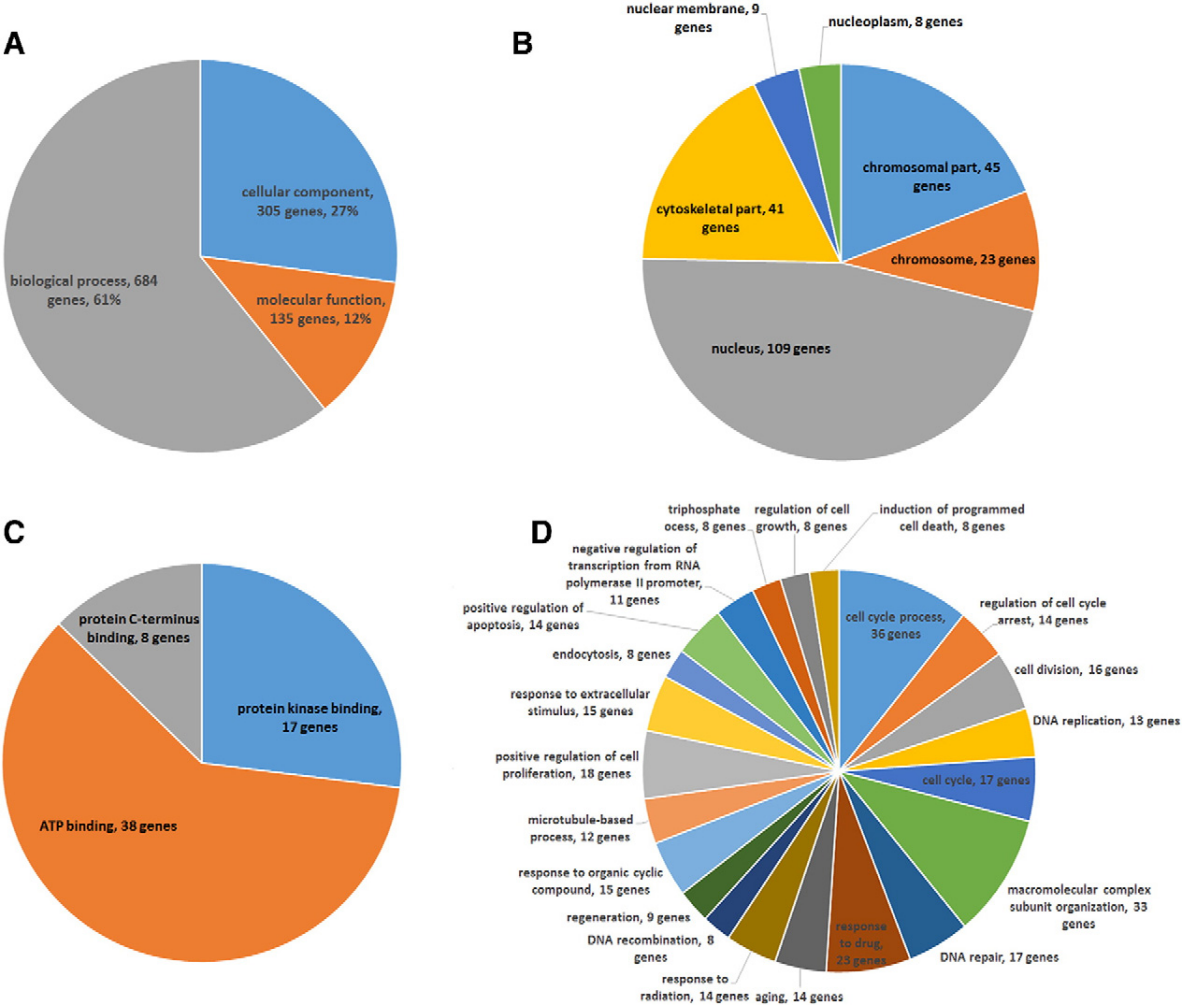
Functional categories of the genes showing significant > 2 fold expression change in DPCs at 24 h relative to control. A, Biological categories of the transcripts were assigned with GoElite (http://www.genmapp.org/go_elite) analysis software and further subdivided by category; B, Cellular component; C, molecular function; and D, biological process. Only GoTerms categories with greater than 7 transcript members are included.

Raw data were exported to GeneSpring GX12 and signals for each replicate spot were background corrected and normalized using Lowess transformation. Log2 fluorescent intensity ratios were generated for each replicate spot and averaged. Genes that were differentially expressed (> 2.0 fold) in the SAHA group relative to control were identified after passing a t-test (p < 0.05), post-hoc test (Storey with bootstrapping) with a corrected q value of 0.05 (Fig. 1). Genes in the expression datasets were first ‘ranked’ based on Log2 values from the highest to lowest for both groups at both time points, prior to hierarchical clustering being used to group gene expression in each condition using the default settings in Genespring GX12. Gene Ontology (GO) was evaluated using Go-Elite (http://www.genmapp.org/go_elite) [6], which is designed to identify a minimal non-redundant set of biological Ontology terms to describe a particular set of genes (Fig. 2).

### 2.5 Validation of microarray data by quantitative RT-PCR analysis

In order to validate the microarray, isolated RNA was converted to single-stranded cDNA using the TaqMan™ reverse transcriptase kit and 50 μM random hexamers (Life Technologies) and the concentrations determined spectrophotometrically (Nanodrop 2000, Thermo Fisher Scientific). A range of transcripts was selected based on their levels of differential regulation. The q-RT-PCR analysis was performed for rat genes using specific primers (Invitrogen, Life Technologies, Thermo Scientific, Paisley, UK) for *Dab1*, *Gsta4*, *Hist1h1b*, *Ska1*, *Epha3*, *Krt18*, *Mal2* and *Rasgrp13*. Primers for each selected gene were designed using Primer3Plus tools (http://primer3plus.com/). Sequence identity of the amplicon was confirmed with NCBI Basic Local Alignment Search Tool (blast) software (http://blast.ncbi.nlm.nih.gov/Blast.cgi)Synthesized cDNA was amplified and PCRs performed using the Applied Biosystems 7500 Fast Real-Time PCR thermal cycler (Applied Biosystems), being subjected to a designated number of amplification cycles (40 cycles), where a typical cycle was 95 °C for 3 s and 60 °C for 30 s. Real-time PCR data were normalized to β-actin. The upregulated and downregulated transcripts assayed by RT = PCR were consistent with the results obtained from DNA microarray (Table 1).

## 3 Discussion

In this report we describe detailed technical methods to reproduce the high-throughput gene analysis using the whole rat genome oligo microarray kit (Agilent, G2519F, Ireland) for mineralizing primary DPC cultures in the presence and absence of a HDACi. The validated gene expression data demonstrates the relevant expression profile signature of differentially expressed genes during mineralization and identifies several transcripts not previously associated with this process. This data can contribute to future investigations examining transcriptional changes that promote tissue repair processes in the dental pulp.

## Figures and Tables

**Table 1:** A comparison of microarray and quantitative RT-PCR for selected genes at 24 h and 14 days.

Gene	Fold change cDNA microarray (24 h)	Fold change RT-PCR (24 h)
Dab1	4.13	5.17
Gsta4	8.21	14.5
Hist1h1b	− 7.83	− 13.0
Ska1	− 97.2	− 72.5

Gene	Fold change cDNA microarray (14 days)	Fold change RT-PCR (14 days)
Epha3	− 2.4	− 2.06
Krt18	− 2.8	− 2.65
Mal2	2.83	2.73
Rasgrp13	2.33	2.32
